# Children’s Nutritional Rehabilitation Program in Beira, Mozambique: A Retrospective Study

**DOI:** 10.4269/ajtmh.20-0297

**Published:** 2021-09-27

**Authors:** Serena Calgaro, Valentina Isidoris, Cristian Girotto, Kajal Chhaganlal, Jorge Moiane, Giovanni Putoto, Liviana Da Dalt, Daniele Trevisanuto, Giovanna Verlato, Damiano Pizzol

**Affiliations:** ^1^ Department of Woman’s and Child’s Health, University of Padova, Padova, Italy;; ^2^ Operational Research Unit, Doctors with Africa CUAMM, Beira, Mozambique;; ^3^ Operational Research Unit, Doctors with Africa CUAMM, Padova, Italy;; ^4^ Center for Research in Infectious Diseases, Faculty of Health Sciences, Catholic University of Mozambique, Beira, Mozambique

## Abstract

Malnutrition is still a major public health issue in sub-Saharan Africa and Mozambique. The main aim of this study was to evaluate the adherence to the nutritional rehabilitation program (NRP) and its impact on the growth of malnourished children in Beira, Mozambique. The secondary aim was to verify the prevalence of HIV infection in malnourished children at the time of admission to the NRP. A retrospective observational study in Beira Central Hospital and 10 health centers in Beira, Mozambique, was conducted. All children 0 to 5 years of age with acute malnutrition admitted to the outpatient services of the NRP from March 2016 until February 2017 were included in the study. A total of 1,231 children with the following characteristics have been enrolled: 58% female; 33% severely malnourished; and 16.5% HIV-positive. Of the 198 (21.7%) children who completed the program, 177 (89.4%) recovered from malnutrition and 21 (10.6%) did not. Ten (1.1%) were hospitalized and 706 (77.2%) dropped out of the program. Among children who completed the program, the median weight-for-length and weight-for-height z-scores at admission were ≥ −3 and < −2; at discharge, these median z-scores were ≥ −1 (*P* < 0.001). Children with HIV infection and who were male had a higher prevalence of severe acute malnutrition (*P* < 0.001). Weight gain was found to be significant after 23 days (*P* = 0.004) of consuming supplements (ready-to-use therapeutic food). A diagnosis of the degree of malnutrition was accurate at admission for 70.5%; at discharge, this diagnosis was accurate for 67.2%. The NRP seems to be successful if correctly followed, even if it is limited by adherence problems. However, its effectiveness requires further investigation.

## INTRODUCTION

Globally, malnutrition is considered a major public health issue in low-income countries. According to sustainable development goals, by 2025, stunting and wasting in children younger than 5 years of age should end, and by 2030, all forms of malnutrition should end.^1^ In 2020, approximately 191 million children younger than 5 years were malnourished worldwide.^2^ The majority of malnourished children live in low-income and lower-income to middle-income countries; specifically, 92% of wasting and 91% of stunted children live in these countries.^2^ Asia seems to be the most affected region.^2^ However, alarming data have emerged from Africa indicating that it is the only region where the number of stunted children has increased from 2000 to 2019; in 2019, the rate of wasting children younger than 5 years was 6.4%.^2^

In Mozambique, according to the National Institute of Statistics, children represent 52% of the national population.^3^ From 2009 to 2013, 17% of children had low birth weight and only 11% of children from 6 to 23 months were receiving the minimum recommended acceptable diet.^4^ Moreover, moderate and severe acute malnutrition are major reasons for accessing healthcare services in Beira.^5^ In Mozambique, approximately 44% of children younger than 5 years have chronic malnutrition (in Sofala 39%) and 4% have acute malnutrition.^6^

In Mozambique, to ensure the effective treatment and nutritional rehabilitation of children with acute malnutrition, the Ministry of Health established the nutritional rehabilitation program (NRP) in 2010. It includes community involvement, inpatient malnutrition treatment, outpatient malnutrition treatment, nutritional supplementation, and nutrition education including cooking demonstrations.^7^

An additional worsening and mutual risk factor is HIV/AIDS because of the high micronutrient requirements during all stages of the disease. The WHO guidelines advise that children with HIV should increase their energy intake up to 150% of the recommended daily allowance and their micronutrient requirement up to five-times the normal intake.^7^ In 2019, 1.8 million children younger than 15 years worldwide^8^ had HIV; in Mozambique alone, there were 140,000 children younger than 15 years with HIV in 2018.^9^ A study performed in Beira between 2015 and 2017 showed that HIV exposure to children was the main reason for accessing health services.^5^ The prevalence of HIV infection increases with age; in Beira, women older than 24 years have a higher prevalence of HIV infection than adolescents.^10^ Although the connection between HIV and malnutrition is well-known, insufficient data are available in the Sofala province of Mozambique.

The main aim of this study was to evaluate the adherence to the NRP and its impact on the growth of children 0 to 5 years of age with acute malnutrition in Beira, Mozambique, after its introduction. The NRP is a new national protocol of nutritional rehabilitation. The secondary purpose of this study was to evaluate the prevalence of HIV infection in malnourished children.

This is the first study to describe the adherence to and the impact of the NRP in Mozambique. We believe that an assessment of the effective implementation and impact of the NRP will help to identify specific weaknesses, thereby allowing us to correct them and improve the outcomes of children, particularly those with comorbidities such as HIV infection, which may worsen their clinical situation.

## MATERIALS AND METHODS

### Study design.

We performed a retrospective, observational study of the adherence to the recommendations of the outpatient services of the NRP and the impact of the NRP on the growth of children 0 to 5 years of age treated for acute malnutrition in Beira (Mozambique) from March 2016 to February 2017.

### Setting.

Beira is a city located in Sofala province, which is in the central area of Mozambique. The population is approximately 550,000. Beira Central Hospital and 10 health centers (HCs), distributed within the area comprise the health facilities of the NRP for children with acute malnutrition. The Beira Central Hospital is a multispecialist 1,000-bed referral hospital for the entire Sofala province comprising approximately 2 million people. Its nutritional rehabilitation unit serves as stabilization center for patients referred from HCs with complications. It consists of 22 ordinary and 2 semi-intensive care beds, and the medical team comprises one pediatrician, two general physicians, four or five medical students, and two nurses. It also has an outpatient service (OS) department for follow-up after discharge. The HCs involved in the NRP are Munhava, Macurungo, Mascarenha, Chamba, Cerâmica, Ponta Gêa, Nhaconjo, Manga Loforte, Chota, and Nhagao. The HCs have an OS department managed by one nurse. The clinics have a scale, statimeter, and tape measure to obtain anthropometric parameters such as weight, height, and the mid upper arm circumference (MUAC).

### Nutritional Rehabilitation Program.

The inclusion criteria of the NRP are children with a diagnosis of acute malnutrition with or without medical complications, diagnosis of acute malnutrition obtained through anthropometric measurements such as the weight-for-length z-score (0–2 years) or weight-for-height z-score (older than 2 years) < −2 and/or MUAC < 12.5 cm and/or clinical criteria such as edema, signs of thinness, or signs of rapid weight loss.^7^

Malnourished children older than 6 months without medical complications (bilateral edema, seizures, coma, lethargy, hypoglycemia, hypothermia, severe dehydration, lower respiratory tract infection, high fever, severe anemia, intractable vomiting, lack of appetite, skin changes, signs of vitamin A deficiency) are treated in the OS department of the HCs. The treatment of outpatient children is based on the use of routine medications (such as antibiotics, anthelmintics, antimalarials, vitamins, and trace elements) and the administration of ready-to-use therapeutic food (RUFT) or *Alimento Terapêutico Pronto para Uso*, which is food enriched with vitamins and minerals that has high energetic density and is designated for the treatment of severe acute malnutrition. The most used RUFT is Plumpy Nut (Table 1). In the case of moderate acute malnutrition, a blend of corn and soy flour-enriched formula with vitamins and minerals is used. At the HCs, regular evaluations of these children are performed (usually every 15 days), and the average outpatient program lasts 2 months.^7^

**Table 1:** Ready-to-use therapeutic food (Plumpy Nut) nutritional information (per 100 g)

Macronutrients	Vitamins	Minerals
Energy: 545 kcal	Vitamin A: 910 μg	Calcium: 320 mg
Protein: 13.6 g	Vitamin D: 16 μg	Phosphorus: 394 mg
Fat: 35.7 g	Vitamin E: 20 mg	Potassium: 1,111 mg
	Vitamin C: 53 mg	Magnesium: 92 mg
	Vitamin B1: 0.6 mg	Zinc: 14 mg
	Vitamin B2: 1.8 mg	Copper: 1.78 mg
	Vitamin B6: 0.6 mg	Iron: 11.53 mg
	Vitamin B12: 1.8 μg	Iodine: 110 mcg
	Vitamin K: 21 μg	Sodium: < 290 mg
	Biotin: 65 μg	Selenium: 30 μg
	Folic acid: 210 μg	
	Pantothenic acid: 3.1 mg	
	Niacin: 5.3 mg	

Children younger than 6 months or with medical complications are hospitalized and the program is divided into four phases: stabilization and transition; admission to Beira Central Hospital for approximately 10 days; rehabilitation and monitoring; and admission to the OS department for 19 weeks.^7^

Children treated by the OS department can be discharged when they meet the following criteria: weight-for-length/weight-for-height z-score ≥ −1 at two consecutive follow-up visits, presence of good appetite, and the possibility of eating food at the family home. Children who meet all these criteria at discharge are considered “recovered” from malnutrition.^7^ Children who are discharged but do not meet these criteria either died during treatment or did not fully recover after 4 months of treatment without any evident cause of the lack of response. Those children are considered “not recovered” from malnutrition. Children who do not attend more than two consecutive follow-up visits before meeting the criteria are considered to have dropped out of treatment.^7^

### Patients.

All children 0 to 5 years of age admitted to the OS department (both of the CHB and of the HCs) in Beira for acute malnutrition were included in the study.

### Outcome measures.

The primary outcome measures were the percentage of children who completed the program (recovered and not recovered from malnutrition) and the differences between the median weight-for-length and weight-for-height z-scores at admission and those at discharge from the NRP to evaluate the impact on growth. Normal nutritional status was indicated by weight-for-length and weight-for-height z-scores ≥ −1; mild malnutrition was indicated by weight-for-length and weight-for-height z-scores ≥ −2 and < −1, respectively; moderate malnutrition was indicated by weight-for-length and weight-for-height z-scores ≥ −3 and < −2, respectively; and severe malnutrition was indicate by weight-for-length and weight-for-height z-scores < −3).^7^

The secondary outcomes measures were the prevalence of HIV infection in children in the study and the prevalence of severe acute malnutrition in children with HIV.

### Data collection and statistical analysis.

All the variables were retrospectively extracted from individual data routinely collected in the medical records by trained health staff members. Sex, age, HIV infection, weight, length (0–2 years) or height (older than 2 years), weight-for-length and weight-for-height z-scores, percentage of weight-for-length and weight-for-height z-scores correctly interpreted, MUAC, types of supplements used, and outcomes of the patients were evaluated.

Data management was performed using Microsoft Excel 2013. Statistical analysis was performed using R version 3.4.1. Data are expressed as the median and interquartile range (IQR; quartiles I–III). Comparisons of medians were performed using the Wilcoxon test. The Fisher test was applied when the expected values were ≥ 5. Furthermore, the χ^2^ test was used to compare categorical variables. Because of the large amount of missing data, only patients with available data were considered for each outcome.

## RESULTS

### Primary and secondary outcomes.

We enrolled 1,231 children (708 [58%] were female and 513 [42%] were male) with a median age of 14 months (IQR, 10–19). Clinical data are reported in Table 2.

**Table 2:** Children’s data regarding sex, age, HIV status, weight, height and mid upper arm circumference

Sex, n (%) (N = 1,221)	Female	708 (58%)
	Male	513 (42%)
Age, median (IQR)	Female	14 months (10–19 months)
	Male	14 months (10–19 months)
HIV infection, n (%) (N = 952)	Negative	795 (83.5%)
	Positive	157 (16.5%)
Weight, median (IQR)	At admission (n = 1,227)	6.9 kg (6.1–7.2 kg)
	At discharge (n = 659)	7.5 kg (6.7–7.8 kg)
Height, median (IQR)	At admission (n = 1,174)	71 cm (67–76 cm)
	At discharge (n = 644)	72 cm (68.76 cm)
Mid upper arm circumference, median (IQR) (N = 635)	At admission: 12 cm (11–12 cm)	

**Figure 1.**
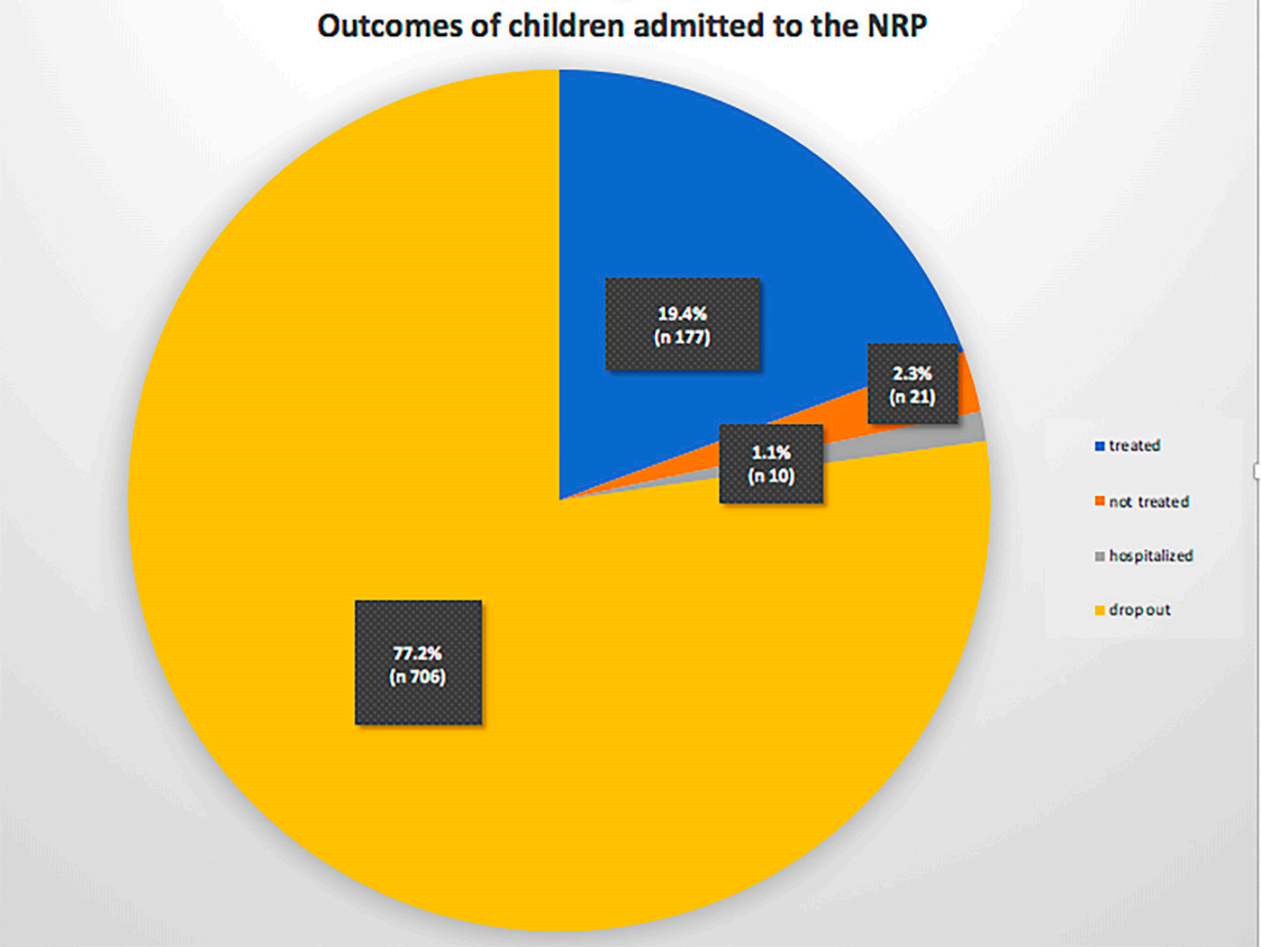
Outcomes of children admitted to the nutritional rehabilitation program (NRP). This figure appears in color at www.ajtmh.org.

Data of the outcomes showed that 198 (21.7%) children completed the program; of these, 177 (89.4%) recovered and 21 (10.6%) did not. Ten (1.1%) were hospitalized and 706 (77.2%) dropped out of the program. Outcomes data were unavailable for 317 children (Figure 1).

**Figure 2.**
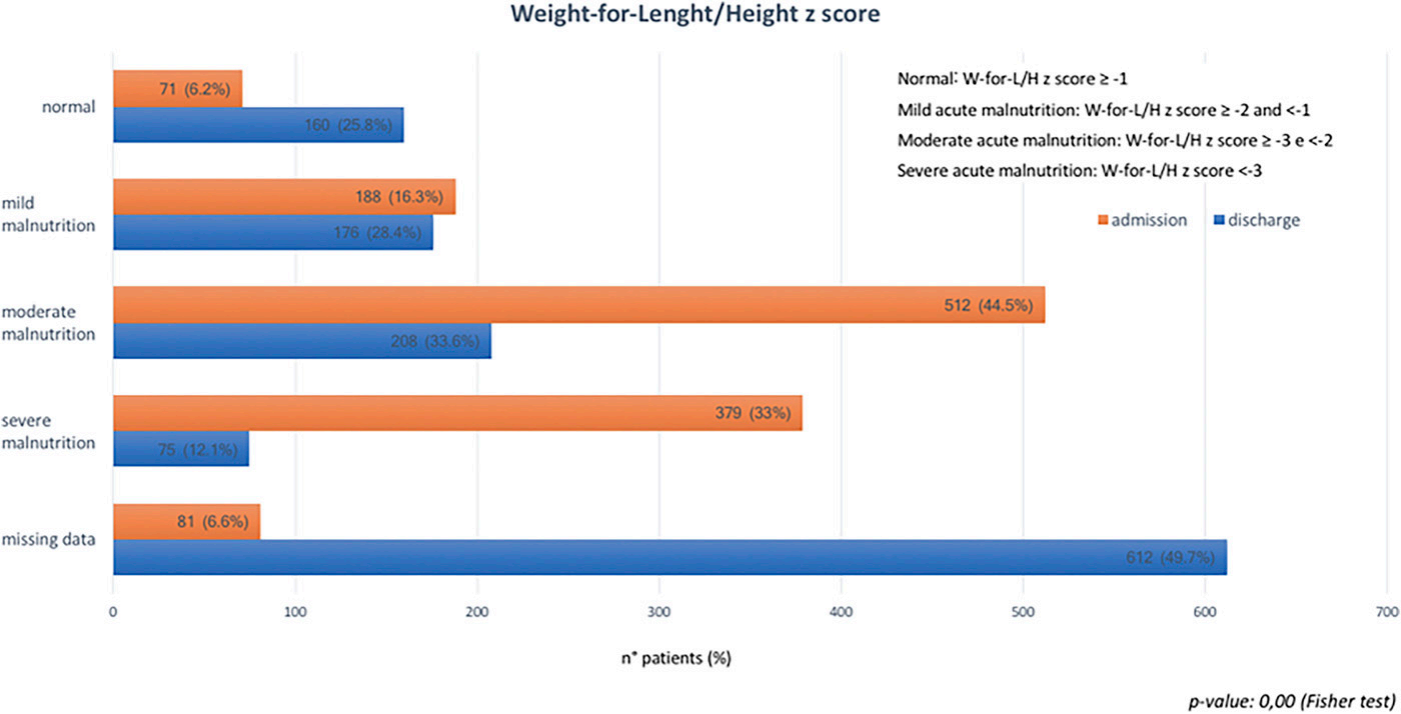
Weight-for-length and weight-for-height z-scores at admission and discharge. This figure appears in color at www.ajtmh.org.

Figure 2 shows data regarding the nutritional status evaluated with anthropometric parameters at admission and at discharge. At admission, 512 (44.5%) had moderate and 379 (33%) had severe acute malnutrition; 71 (6.2%) children had weight-for-length/weight-for-height z-scores ≥ −1. At admission, the median weight was 6.9 kg (IQR, 6.1–7.2 kg) and the median length/height was 72 cm (IQR, 68–76 cm). The median MUAC at admission was 12 cm (IQR, 11–12 cm) (Table 2). Among children who completed the program (*N* = 198), the median weight-for-length and weight-for-height z-scores at admission were ≥ −3 and < −2; at discharge, they were ≥ −1 (Wilcoxon test, *P* < 0.001). At discharge, the median weight was 7.5 kg (IQR, 6.7–7.8 kg), and the median length/height was 72 cm (IQR, 68–76 cm) (Table 2).

Of all the children who underwent the HIV test (*N* = 952), 157 (16.5%) were HIV-positive. Children with HIV had a higher prevalence of severe acute malnutrition: 49% of HIV-positive children and 32% of HIV-negative children had severe acute malnutrition (chi-square test, *P* < 0.001) (Table 3).

**Table 3:** Weight-for-length and weight-for-height z-scores of HIV-positive and HIV-negative children at admission

		Weight-for-length and weight-for-height z-scores at admission				
		≥ −1	≥ −2 and < −1	≥ −3 and < −2	< −3	
HIV status	Negative, n (%)	33 (4)	129 (17)	351 (47)	241 (32)	*P* < 0.001 (chi-square test)
	Positive, n (%)	2 (1)	16 (12)	54 (38)	70 (49)	
	No test performed, n (%)	12 (13)	14 (15)	44 (46)	25 (26)	

### Other results.

Males had a higher degree of malnutrition than females (Fisher test, *P* < 0.001). The most used supplement was RUFT (Plumpy Nut), which was used by 1,059 (87.8%) of children; however, a blend of corn and soy flour was used for 107 (8.9%) of children. Data were unavailable for 25 children. The median time during which supplements were consumed was 16 days (IQR, 7–28 days). Weight gain was found to be significant after 23 days (Fisher’s test, *P* = 0.004).

**Figure 3.**
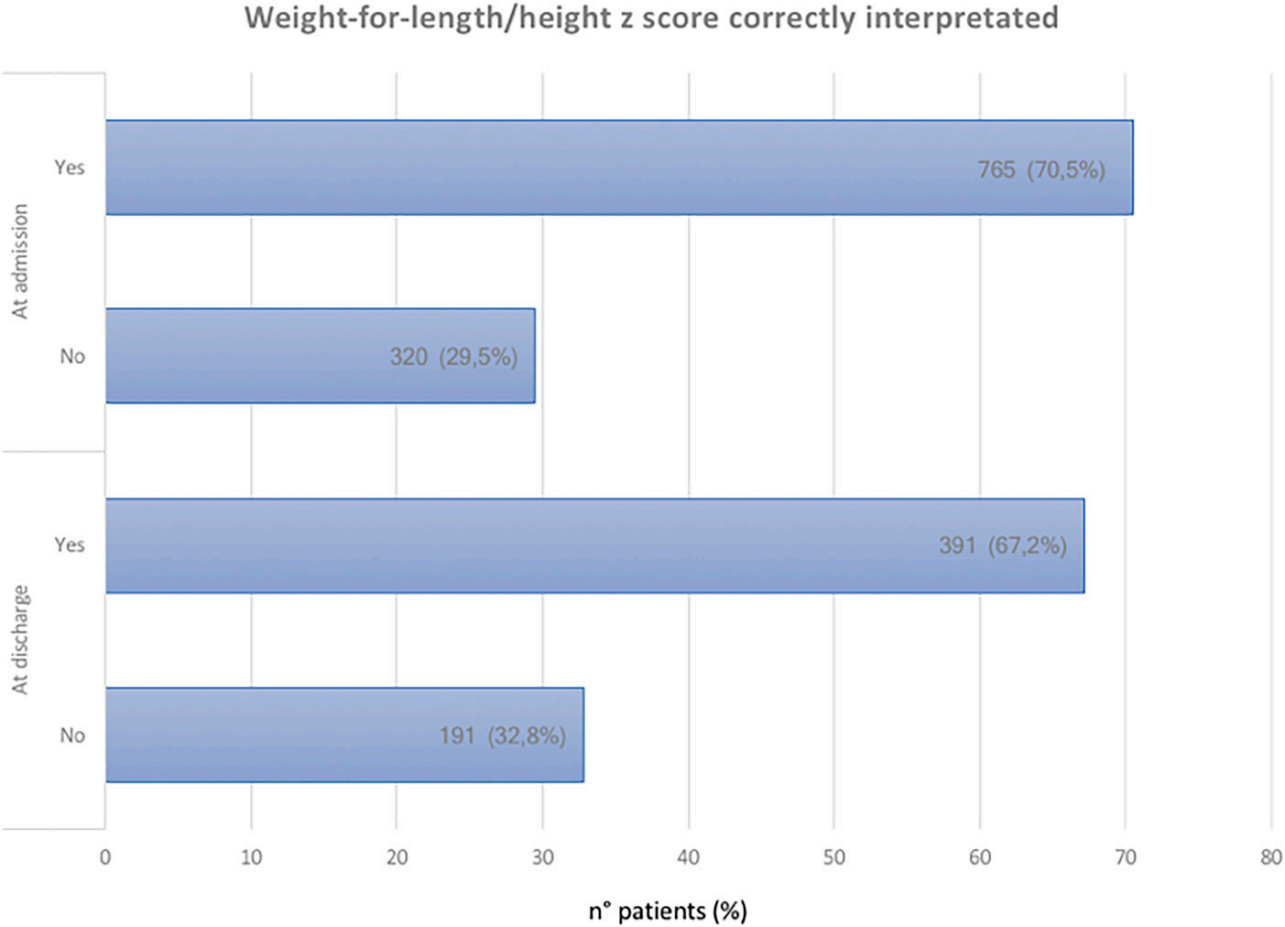
Weight-for-length and weight-for-height z-scores correctly interpreted. This figure appears in color at www.ajtmh.org.

We retrospectively assessed the accuracy of the diagnosis of the degree of malnutrition performed by health professionals at HCs. We found that it was accurate for 765 (70.5%) children and 391 (67.2%) children at admission and discharge, respectively (Figure 3).

## DISCUSSION

During our study, we are able to affirm that in Mozambique, acute malnutrition is a crucial and emerging public health issue. In particular, the rate of severe acute malnutrition is dramatic and probably reflects the unsuitableness of their health services and delays in access to care services despite the malnutrition, child care, and health education programs created by the Mozambique government. These programs include nutritional supplementation interventions, school health and agriculture programs, community prevention campaigns, and nutrition and food security plans.^11^

To obtain the planned results, adherence to the ministerial programs is fundamental. During this study we first assessed adherence to the NRP. Data showed that a high rate of patients, approximately 75%, dropped out of the program before its conclusion. This fact has already been reported in the literature. Takyi et al. confirmed poor adherence to NRPs for children in Ghana and argued that greater efforts must be made to promote adherence.^12^ A study performed in Ethiopia involving HIV-positive adult patients showed poor adherence to nutritional programs and that the only sociodemographic variable with a significant effect on adherence is the educational status.^13^ Therefore, further studies are needed to evaluate the factors that negatively affect adherence to health programs for children.

Despite the high level of missing data at discharge and the high number of patients who dropped out, the outcomes of children who completed the NRP indicated that the majority improved their nutritional status. Therefore, the nutritional program seems to work if adhered to; however, there is not yet enough information to evaluate its effectiveness in the long term. The effectiveness of NRPs in developing countries have been confirmed by other studies performed in Ghana and Pakistan.^12^^,^^14^

The association between HIV and underweight, stunting, and wasting has been described in the literature.^15^^–^^17^ Furthermore, it is known that among malnourished children, those living with HIV have a higher rate of severe malnutrition than HIV-negative children.^15^^,^^18^ Our study results confirmed that HIV-positive children have a higher prevalence of severe malnutrition (nearly 50% of our sample) than HIV-negative children (Table 3). Similar results were described by Poda,^15^ who reported that the prevalence rates of severe malnutrition were 31% and 5% among HIV-positive and HIV-negative children. Although the pathophysiological mechanism is not yet clear, the most probable is the correlation between HIV and malnutrition.^16^^–^^24^ Patients with HIV need to consume a higher amount of calories; however, the state of malnutrition worsens the clinical situation of patients with HIV. Therefore, the nutritional assessment is important for preventing malnutrition in these patients. In 2018, Marotta et al. reported that implementation of task-shifting from clinical officers to maternal and child nurses in the Beira district increased regular nutritional assessments and contributed to improving the global effectiveness of care for HIV-infected children.^25^

Another interesting outcome was that male children are more malnourished than female children.^26^^,^^27^ The reasons for this are still unknown, but it may be related to bias, the greater biological fragility of males, different food practices, or different cultural factors.

The enteral supplement used most often was RUFT (Plumpy Nut), which is in accordance with the recommendations of the NRP. An innovative and interesting discovery regarding the length of treatment was that the RUFT supplement must be consumed for at least 23 days to ensure a significant increase in weight. This result is in contrast to the results described by studies conducted in Uganda and Nigeria, where the diets were supplemented with similar RUFT and weight gain was noticed after only 2 weeks.^26^^,^^27^ However, these results were not comparable with ours because children with severe acute malnutrition were excluded from these trials. Considering these results, the high rate of drop out, and the possible abandonment of the program before completing 3 weeks, it is crucial to guarantee at least 3 weeks of adherence to the program to ensure a high probability of improving the nutritional status of admitted children.

A dramatic discovery was that for approximately one-third of children, both at admission and at discharge, medical staff members incorrectly diagnosed the degree of malnutrition and misinterpreted the anthropometric parameters, thus reflecting that data recording was not performed consistently or properly. The OS department of HCs are managed by one nurse who has to attend to a large number of children every day. This work overload that occurs can result in diagnostic failure. Similarly, sometimes a lack of qualified and adequately paid staff members can affect the data accuracy. Even the use of paper records can facilitate the accumulation of errors in transcriptions or the misinterpretation of written records, which are easily degradable. Therefore, it would be appropriate to introduce both appropriate specific training courses for staff members and programs for monitoring the accuracy and quality of the medical records in the programs.

This study had some limitations. The missing data greatly reduced the sample size, thereby affecting our ability to assess the impact on growth. This reflects the relevant problems associated with data accuracy and data management in developing countries. Another important limitation was the lack of evaluation of the factors that influence the adherence to the NRP by children and caregivers.

Nonetheless, to the best of our knowledge, this is the first study performed in Sofala province and one of few studies performed in Mozambique to assess adherence to nutritional delivery services after the introduction of the NRP and the association between the nutritional status and HIV status of children.

In conclusion, malnutrition, especially in HIV-positive children, is an important problem in Mozambique. This study highlights how the NRP seems to be effective if correctly followed. However, the effectiveness of the NRP is limited by the high abandonment rate and possible difficulties with therapeutic adherence.

Based on these results, it is necessary to establish educational strategies addressing the population and health care professionals to improve the quality and effectiveness of services, encourage better adherence to existing health programs, and ensure proper data collection.
